# Rising Incidence and Mortality of Colorectal Cancer in Young African Adults: Need for a Better Care Plan

**DOI:** 10.7759/cureus.85866

**Published:** 2025-06-12

**Authors:** Francis I Anazodo, Chinedu G Ezeah, Catherine C Eleje, Kamsiyochukwu A Ezeani, Faizah Alabi, Ebunolorun I Ayo, Chiamaka P Agu, Izuchukwu F Okpalanwaka

**Affiliations:** 1 Department of Biochemistry, Cancer Biology, and Immunology, Medical College of Georgia, Augusta University, Augusta, USA; 2 Oncology Research Group, Tropical Medical Oncology Awareness Foundation, Lagos, NGA; 3 Department of Pharmaceutical Chemistry, University of Lagos, Lagos, NGA; 4 Department of Pharmaceutical Technology and Industrial Pharmacy, University of Nigeria, Nsukka, NGA; 5 Department of Medicine, University of Nigeria, Enugu, NGA; 6 Department of Immunotherapeutics and Biotechnology, Texas Tech University Health Sciences Center, Abilene, USA; 7 Department of Nutritional Sciences, Auburn University, Auburn, USA; 8 Child Study Center, Yale School of Medicine, New Haven, USA

**Keywords:** cancer epidemiology, cancer mortality, cancer screening strategy, colorectal cancer, fecal occult blood test

## Abstract

Colorectal cancer (CRC) continues to be a significant cause of cancer-related deaths worldwide. Global advancements in health and improved quality of life have increased expectations for reduced incidence and mortality of cancers, including CRC. CRC in adults under the age of 50 years is referred to as early-onset CRC. The incidence of CRC among African youths is increasing. Genetic and lifestyle factors, limited awareness of the disease's risk factors, and restricted access to screening and early diagnostic services can predispose young African adults to CRC. Understanding the current state of diagnosis and management of CRC in young African adults is essential for developing treatment modalities that improve patient outcomes. In many parts of Africa, diagnostic capacity and treatment infrastructure remain underdeveloped, leading to late-stage detection and poor outcomes. Although some clinical guidelines tailored to low-resource settings have been introduced, their adoption and the outcomes of their application remain underreported. This review evaluates existing literature to summarize the current state of CRC diagnosis and management in Africa. It examines emerging trends, systemic barriers, and actionable strategies for improving outcomes among young adults. We then propose multi-pronged approaches that adapt to local needs, offering effective management considerations for CRC in young African adults.

## Introduction and background

Colorectal cancer (CRC) is an overgrowth of large intestinal cells that begins in the colon or rectum. CRC is the third most commonly diagnosed cancer globally, but it represents the second leading cause of cancer-related deaths [1,2]. Most CRC deaths are due to cancer metastasizing to other organs of the body [3]. CRC diagnoses in the early stage of the disease significantly improve the survival rate. The highest incidence of CRC occurs in countries with a high Human Development Index (HDI) [4]. Although the incidence of CRC in people over the age of 50 years has steadily decreased over the past few decades, in contrast, the incidence in younger adults below 50 years has also increased [5,6]. The increasing incidence of CRC in younger adults, termed early-onset colorectal cancer (EOCRC), has been attributed to genetics, obesity, increased alcohol use, changes in diets, etc. [7]. It was estimated that in 2030, EOCRC will be the leading cause of cancer-related deaths in people below 50 globally [8]. However, CRC has become the primary cause of cancer-related deaths in men under the age of 50 [9]. This is likely as a result of the fact that younger CRC patients mostly present with advanced disease at diagnosis [10]. Many countries have adjusted the recommended screening age for CRC to reflect the recent increase in the occurrence of the disease in younger adults. In 2021, the US Preventive Services Task Force recommended that the screening for CRC should start from 45 instead of the previously recommended age of 50 [11]. In the US, approximately 13% of all estimated CRC diagnoses are expected to occur in adults younger than 50 years. EOCRC in Africa has received less attention, even though Africa has a higher burden of EOCRC compared to many other regions of the world [12]. The rising EOCRC in Africa, along with its associated mortality, poses significant public health issues. Hence, strategies aimed at addressing this concern are urgently needed. The lack of advanced healthcare infrastructure and care plans for this category of patients in Africa necessitates urgent action to improve outcomes for EOCRC patients. In this review, we examine the challenges of EOCRC in Africa and explore potential solutions for enhanced management and improved outcomes of the disease. This review will outline strategies that, if adopted, could help advance the diagnosis and management of EOCRC in Africa.

## Review

Epidemiology of CRC in Africa

**Figure 1:**
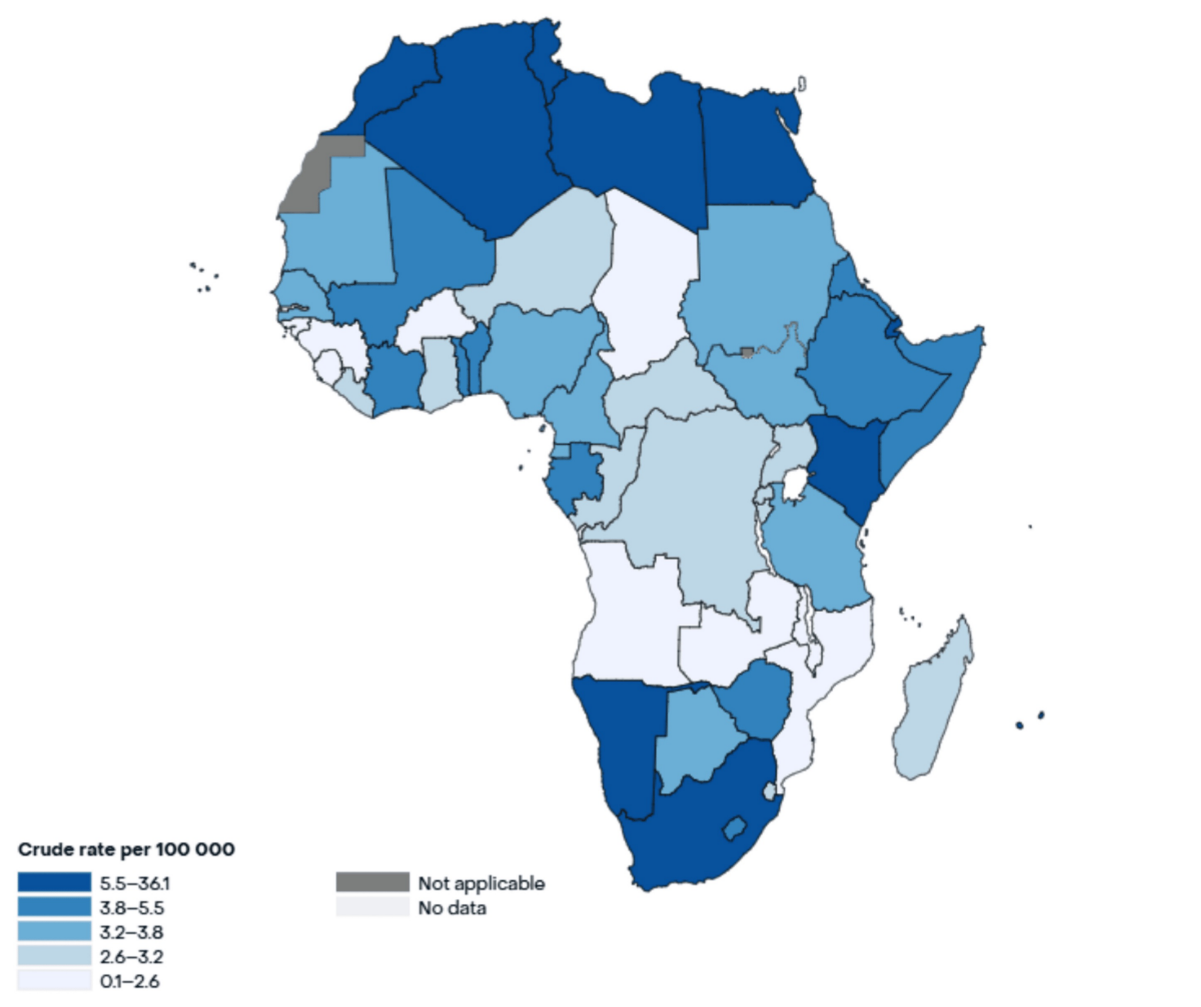
Incidence rate of CRC in Africa. The figure shows the crude incidence rate of colorectal cancer (CRC) in different African countries, with countries in Northern and Southern Africa showing the highest incidence, according to the GLOBOCAN 2022 global database [14].

As of 2022, CRC incidence and mortality rates in Africa were estimated at 8.2 and 5.6 per 100,000 people, respectively, with a notable preference for the male population compared to females (9.1 versus 7.5). The absolute numbers indicated approximately 36,427 cases of colon cancer, 27,645 cases of rectal cancer, and 6,356 cases of anal cancer recorded across Africa, totaling 70,428 CRC cases in 2022 [13]. Figure 1 shows the incidence rate of CRC in Africa [14].

There is a noticeable regional disparity in CRC incidence across Africa, with higher rates reported in Northern and Eastern regions compared to Western and Southern regions [15]. Despite making up only 18.4% of Africa’s population at that time, Northern Africa represented a significant majority of the cases (33.4%). Although the exact causes of this disparity remain unclear, some studies have attributed it to differences in lifestyle and metabolic risk factors. The difference in diet between Northern and Sub-Saharan Africa (SSA) may play a role [16]. In terms of mortality, the majority of CRC deaths occurred in Eastern Africa (30.0%), followed closely by Northern Africa (28.4%). Together, Eastern and Northern Africa accounted for over half of the continent’s CRC incidences and mortalities, at 60.9% and 58.4%, respectively [13].

Africa exhibits some of the highest age-adjusted cancer mortality rates in the world, and the cancer incidence in Africa is projected to double by 2040. Despite facing this inequitable burden of the disease, cancer patients in Africa are significantly understudied throughout the cancer care continuum. Individuals of African descent represent a mere fraction (less than 3%) of the global genomic data available for cancer research and are often the most underserved by developments in precision medicine [17].

The crude incidence of CRC in SSA for both genders was recorded at 4.04 per 100,000 population (4.38 for men and 3.69 for women). Incidence rates increased with age, peaking in Southern Africa, notably in South Africa. The frequency of CRC in SSA was considerably lower than that observed in high-income nations [18]. At the national level, CRC is among the top four most frequently diagnosed cancers in over half (56%) of African countries. Age-standardized rates (ASRs) for incidence and mortality were greater in males than in females. Predictions indicate that new cases will surge by 139.7% (from 70,428 in 2022 to 168,683 in 2050) if current incidence trends continue. Likewise, mortality rates are expected to rise by 155.2% (from 46,061 in 2022 to 117,568 in 2050). Without significant interventions, both the incidence and mortality rates of CRC are projected to rise in Africa and will more than double by 2050 [13].

The rise in age-standardized death rates and disability-adjusted life years (DALYs) due to CRC reflects minimal progress in the standard care, diagnosis, and treatment of CRC, as well as in the primary prevention of modifiable risk factors and the application of secondary prevention methods [19].

National-level investigations have shown notable differences in both the occurrence and death rates of CRC. The ASR per 100,000 individuals was highest in Algeria (17.1%), with Mauritius (16.9%) and Libya (15.9%) following closely behind, while the ASR for mortality peaked in Libya (11.7%), trailed by Algeria (9.4%) and South Africa (8.9%) [13]. If the incidence rate remains unchanged, CRC cases are expected to rise from 70,428 in 2022 to 168,700 by 2050. Likewise, based on the current mortality rate, an estimated 117,600 individuals are projected to die from CRC in 2050, increasing from 46,061 in 2022. These numbers indicate percentage increases of 139.7% for incidence and 155.2% for mortality, respectively. According to studies, the largest absolute increase in both new cases and deaths is expected to happen in high HDI countries, with a projected rise of 30.69% (which translates to an additional 436,184 cases) and a 51.9% increase in deaths by the year 2050. In contrast, the most pronounced relative increases in incidence and mortality are predicted for low HDI nations, with 148.7% and 150.6% growth rates, respectively, underscoring the rapidly changing demographics and lifestyles in these areas [13].

EOCRC in Africa

Although the age-standardized incidence rate of CRC has increased in Africa in the whole population within the last few decades [20], it is the sharp rise in the population below the age of 50 that raises the most concern. The reason for this concern is the fact that CRC has traditionally been found almost exclusively in older adults above 50 years [21]. More studies are needed to understand the full extent of EOCRC incidence in young adults.

**Figure 2:**
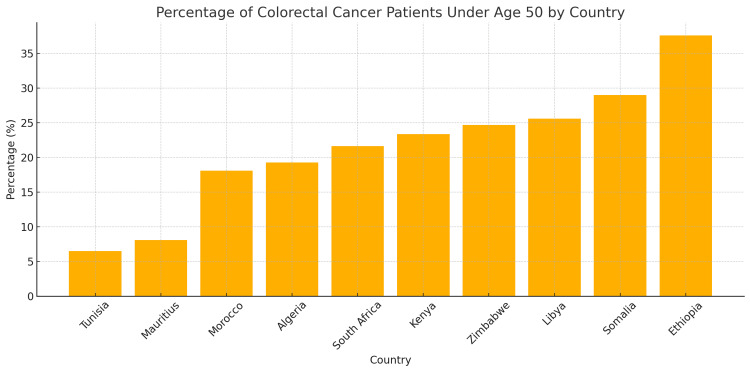
The percentage of colorectal cancer (CRC) patients below the age of 50 years in some African countries. The figure shows the top 10 African countries based on early-onset colorectal cancer (EOCRC) diagnosis, according to 2022 data published by GLOBOCAN [14].

A study by Holowatyj et al., which evaluated the patterns of EOCRC among Nigerian and African American CRC patients in Nigeria and the United States, found that between 1989 and 2017, 60% of Nigerian CRC patients were EOCRC compared to only 13.2% of African Americans. This result suggests the possible combination of genetic, lifestyle, and environmental factors as the major driving force behind the increased incidence of EOCRC in Africa [22]. The top 10 African countries with the highest percentage of EOCRC in Africa are presented in Figure 2 from the data reported in 2022 by GLOBOCAN [14].

**Figure 3:**
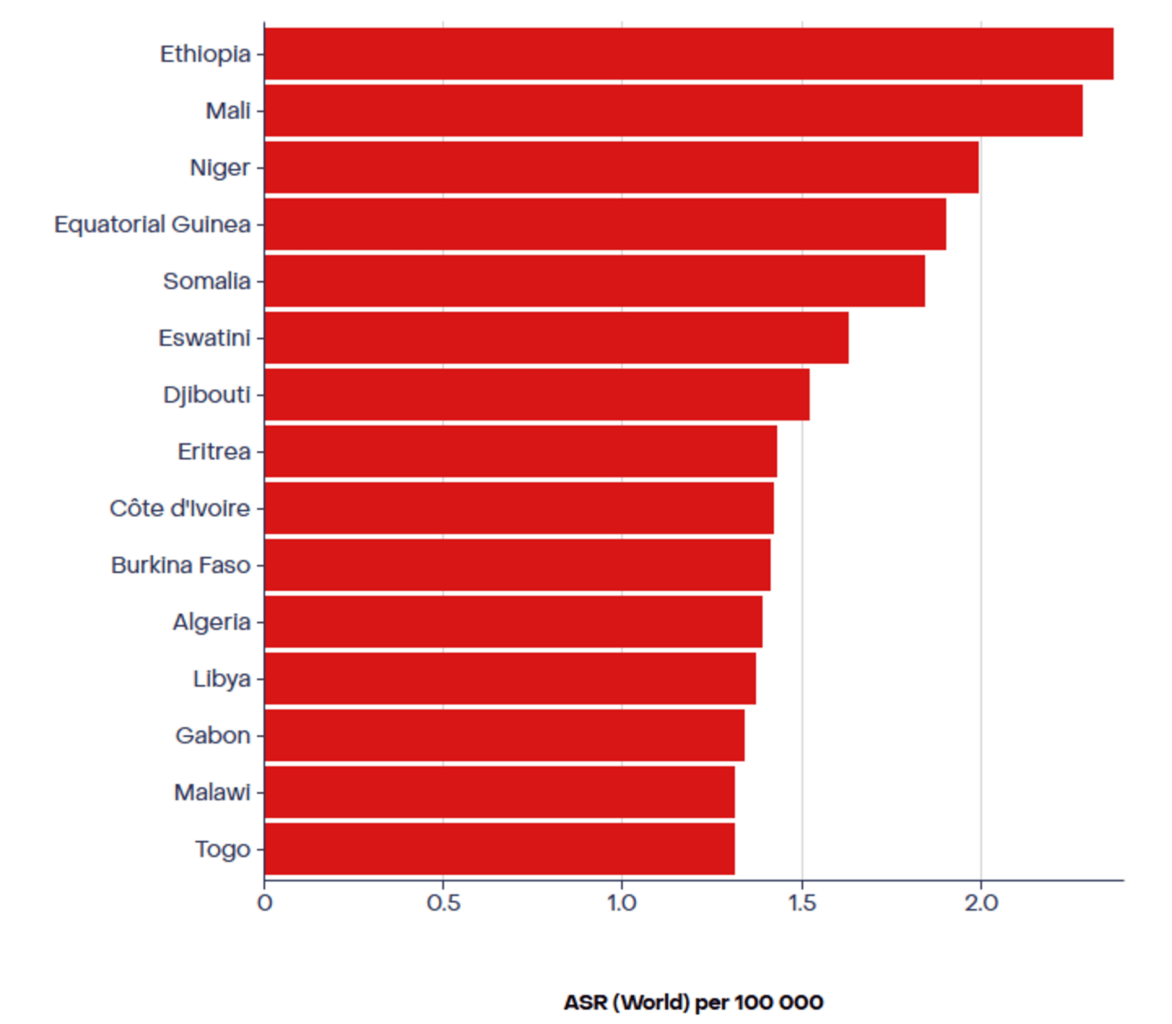
The age-standardized rate (ASR) mortality of colorectal cancer (CRC) patients below the age of 50 years in Africa. The figure shows the ASR mortality of CRC patients below the age of 50 years in African countries with the highest early-onset colorectal cancer (EOCRC) mortality according to 2022 data published by the GLOBOCAN database [14].

Although 50 years has been the global cutoff age for the definition of EOCRC, it is reported that about 25% of all CRC patients in Africa are younger than 40 years [23]. Another study in Ethiopia found that as much as 13% of the CRC cases studied between 2015 and 2018 are in patients aged 30 years or younger [24]. Similarly, a study found that about 22% of the CRC participants are under 40 years of age in Zimbabwe [23]. Another Zimbabwean study reported that 61.5% of patients in cancer registry data from 2012 to 2016 were aged below 50 [25]. This is significantly higher than the data provided by the American Cancer Society, which shows that in 2019, only about 20% of CRC patients were younger than 55 [5]. Only about 10%-12% of CRC diagnoses in the United States are EOCRC, which is similar to the rate found among African Americans as reported by Holowatyj et al. [22]. A study that evaluated the data from 20 European countries reported that only 0.13% of young people aged 20-49 years had CRC between 1990 and 2016 [26]. This suggests that the rate of EOCRC diagnosis is higher in Africa than in the US and Europe. It is worth noting that these data did not take into consideration variabilities and limitations posed by the African Cancer Registry, as some cases are not correctly recorded and accounted for. There is a need to improve the health data management infrastructure, especially in remote areas. Figure 3 shows the mortality rates of patients diagnosed with EOCRC in some African countries as reported from the 2022 GLOBOCAN [14].

While a family history of cancer and inherited genetic syndromes like Lynch syndrome are linked to CRC diagnosis [27], lifestyle and environmental factors are likely the major culprits in the rising cases of EOCRC in Africa. Dietary changes, adoption of western lifestyles among Africans, obesity, sedentary lifestyles leading to more obese populations, and increased alcohol usage are among the possible risk factors [7]. Although these lifestyles are well-established in developed nations, many of these nations have an aging population compared to Africa, with nearly 60% of the African population under the age of 25 [28]. This might explain why Africa has a higher percentage of EOCRC compared to many developed nations.

Current state of CRC diagnosis and management in Africa

Currently, the World Health Organization does not provide a screening guideline for CRC, and the guideline provided by the World Gastroenterology Organization is resource-driven [29]. Many developed nations have updated their CRC screening guidelines to reflect the current trend of CRC incidence in young people. In many developing countries, the issue of EOCRC is hardly a top health priority, especially in African countries that already face numerous other urgent health crises, primarily in the area of infectious diseases. Hence, cancer guideline reviews are not as frequent as they ought to be [30-32].

While it can be said that not much emphasis has been placed on the issue of EOCRC in Africa, the issue of cancer in Africa had previously received regional collective attention in an effort to harmonize and galvanize efforts toward effective diagnosis and management of cancer in Africa. In 2016, the African Cancer Coalition (ACC) was established and brought together cancer experts from 12 SSA countries (Burundi, Democratic Republic of Congo, Ethiopia, Ghana, Kenya, Malawi, Nigeria, Rwanda, Tanzania, Uganda, Zambia, and Zimbabwe). The ACC, alongside the National Comprehensive Cancer Network (NCCN) and the American Cancer Society, developed the NCCN harmonized guidelines for SSA. These harmonized guidelines provide recommendations for the screening and treatment of 32 cancer types in the region, including CRC. This resource-sensitive guideline provides recommendations for practitioners to apply evidence-based strategies in line with the patient's unique needs and existing comorbidities. The recommendations of this guideline were based on published evidence in the region and are updated at least every two years [33,34]. The NCCN harmonized guidelines for SSA have been endorsed as the national standards of care in Ethiopia, Malawi, Nigeria, Tanzania, Uganda, and Zambia, and their utilization in CRC screening and treatment [35]. However, there is not adequate information on the implementation and outcomes of these guidelines, especially among young Africans with CRC.

Current screening and diagnosis of CRC in Africa

The recommendations stipulated as generally available standards of CRC screening on the NCCN harmonized guidelines are colonoscopy, guaiac-based test, flexible sigmoidoscopy, and CT colonography, while fecal immunochemical test and multifaceted stool DNA (mt-sDNA)-based tests are seen as highly advanced care that may be technically challenging to utilize. Choice of a screening modality over another depends on clinical findings and the patient's risk status.

The Ghana National Cancer Steering Committee in 2011 recommended that the fecal occult blood test (FOBT) be used to initially screen patients aged 50-70 years, then followed by endoscopic evaluations when the FOBT is positive [36]. Lussiez et al., however, report that for asymptomatic, average-risk CRC patients in Ghana, about 40% of the physicians recommended colonoscopy for initial screening. In comparison, 26.7% recommended a combination of FOBT and flexible sigmoidoscopy for screening [37].

Theyra-Enias et al. report that the median age of presentation with CRC in one-fourth of the patients assessed in the northern part of Nigeria was 41 years [38]. CRC screening in the country often applies to a combination of modalities (annual FOBT with sigmoidoscopy or double contrast barium enema). Most often, either annual FOBT or colonoscopy every 10 years is used for initial surveillance, then followed by a combination of modalities for diagnosis of suspected cases [39].

In Egypt, there is no national guideline for CRC screening for the general populace, and the clinical practice is to screen only the patients at high risk [40]. Nawwar et al., however, developed a theory-based intervention that employs intervention mapping and suggests the effectiveness of the guaiac fecal occult blood test (gFOBT) [41]. Most often, the screening for CRC in the country involves FOBT, followed by an order for colonoscopy when the initial FOBT is positive [42].

The Cancer Association of South Africa (CANSA) recommends screening patients at risk of CRC from age 50 years using colonoscopy, which is to be repeated every 10 years based on the individual's risk factors. A positive FOBT result is further diagnosed using colonoscopy. Additionally, the South African Colorectal Society recommends fecal immunochemical test as well as flexible sigmoidoscopy for CRC screening and diagnosis, and these modalities are utilized in both private and public health facilities in South Africa [43].

In Zambia, the mean age of CRC diagnosis is 48.6 years. Surgical procedure is the most common mode of diagnosis and treatment employed. Endoscopy and imaging are also utilized in diagnosis, but not as frequently as surgery [30].

Current management of confirmed cases of CRC in Africa

According to the NCCN harmonized guideline for SSA, the recommended management strategies include either surgery, neo-adjuvant, adjuvant, or palliative chemotherapy, radiotherapy, or a combination of these modalities, depending on the clinical presentation and the disease stage [33]. The harmonized guidelines have been endorsed as national standards in Ethiopia, Malawi, Nigeria, Tanzania, Uganda, and Zambia [35].

In Nigeria, Sharma et al. reported the treatment options adopted for the patients assessed. A total of 92 cases out of the 120 cases received surgical intervention. Colostomy accounted for 28.4%, 49.2% underwent radiotherapy while 89 patients received chemotherapy as adjuvant, neo-adjuvant, or as palliative intervention, with each treatment option being dependent on disease stage, tumor molecular make-up, and patient performance status [44]. Nigeria utilizes treatment modalities such as surgery, neo-adjuvant, adjuvant, and palliative chemotherapy, as well as radiotherapy. The preference for a treatment option is based on the recommendations of the NCCN harmonized guideline.

In Ghana, of the 359 CRC cases studied in Accra, most patients (231) had laparotomy, with 225 resections [45].

In Burkina Faso, out of 116 CRC patients diagnosed, 87 (75%) had surgical intervention, with chemotherapy and radiotherapy being used in 37 and 16 patients, respectively [46].

In South Africa, Herbst et al. report that 84% of the patient cohort studied underwent primary surgery, while those who underwent chemotherapy received either a capecitabine regimen or a 5-fluorouracil (5-FU) regimen for early-stage and late-stage CRC, respectively [47].

Due to resource constraints, the surgical procedures practiced in Africa remain traditional open surgery. Cost-effective drug regimens are utilized in chemotherapy, and conventional rather than modern and advanced radiotherapy techniques are employed [48].

Need for a better CRC care plan

The projected increases of 139.7% and 155.2% in the incidence and mortality of CRC by 2050 in Africa, as countries strive to improve their HDI, call for urgent attention [13]. These data are particularly concerning, given that EOCRC is rising rapidly and faster than late-onset CRC [49]. A functional healthcare system includes both the preventive and treatment phases of diseases and follow-up care. The following paragraphs discuss the pressing need for an improved care plan to address the growing incidence and mortality of EOCRC in Africa from prophylactic, therapeutic, and post-treatment perspectives.

Prophylactic care

Awareness, screening, and diagnosis are not just tools but essential pillars for disease prevention, including the early detection and treatment of cancers. Unfortunately, many African healthcare systems have yet to adapt to the management of EOCRC, resulting in poor awareness and low screening rates among young people.

Awareness

CRC is among the top five causes of cancer-related deaths in Africa; however, this has not led to sufficient levels of CRC awareness on the continent [13]. This deficiency in awareness is disturbing as both incidence and mortality rates of CRC are increasing in the younger African population [22]. Governments must urgently raise awareness of CRC and its early-onset dimensions, similar to the ongoing efforts for other cancers such as breast, cervical, and prostate cancers. Key messages should focus on modifiable risk factors for CRC, including smoking, alcohol consumption, sedentary behavior, obesity, and poor nutrition [50]. Additionally, the public should be informed about the familial risks of CRC, encouraging individuals with a family history of CRC or related diseases to seek screening and undergo colonoscopy [51]. Clear age guidelines should be provided regarding when screening is necessary.

Awareness of EOCRC can be raised through social media, universities, sports centers, churches, and hospital visits, all of which are places frequented by the younger African population. However, the number of non-governmental organizations (NGOs) focusing on CRC in Africa is low. CRC-focused NGOs should be established, adequately funded, and supported by international collaborations. These organizations should be present in all African nations or regions and open to volunteering by CRC survivors, especially young adults undergoing treatment, to help increase awareness.

Screening and Diagnosis

The young African population is not being adequately screened for CRC, which can be attributed to several factors, including a lack of awareness among patients and some healthcare providers, poor implementation of relevant oncology data, and unwillingness to follow screening guidelines by some primary care physicians [52]. Additionally, screening and diagnosis are influenced by the local geographical context. In low-income nations, cancer screening and diagnostic disparities are usually obvious between urban and rural dwellers, as many rural settings lack access to good diagnostic and health facilities [53]. Africa has one of the highest global populations of rural dwellers, with many countries having more than half of their total population living in rural settings [54,55]. This shows the need for massive improvements in rural health facilities in Africa. Also, continuing education for healthcare practitioners is essential. While CRC screening for every young patient who walks into clinics for a different health need may place a heavy burden on physicians, screening should be mandatory for those presenting with alarming symptoms, such as unexplained weight loss, iron deficiency anemia, or hematochezia, and for any abdominopelvic complaints [56].

A study identified nurses as important health professionals but reported limited knowledge and reduced sense of competence as some of the reasons affecting the functionality of nurses in the early assessment of individuals at risk for cancer [57]. Nurses and other healthcare attendants who record vital signs should be trained to collect medical histories, including family cancer histories. Alongside proper training, adapting already developed risk assessment tools can help nurses assess patients for EOCRC [58]. Some studies have assessed community pharmacist-led interventions in CRC screening, with subsequent recommendations for continued education to improve pharmacists’ knowledge of CRC screening guidelines. There is a need for such studies in Africa to assess the knowledge and role of pharmacists in CRC screening, including the effectiveness of using community pharmacies as points of contact for home-based CRC screening tests [59,60]. The above suggestions primarily focus on improving opportunistic screening for EOCRC in Africa. However, a more organized CRC screening program is also needed to target specific populations, including the young [61]. While a low healthcare workforce and the urgent need to address communicable diseases in parts of Africa may be limiting factors, a population-based study in Nigeria showed that immunochemical CRC screening tests are feasible and acceptable to asymptomatic individuals with a low average risk [62].

According to most international guidelines, the recommended age for CRC screening has decreased from 50 to 45 years. For instance, the American College of Gastroenterology recommends screening at 45 for Black men and women at average risk. Adequate evidence is lacking regarding whether most African countries have adopted and implemented the new age recommendation. Moreover, extra studies are needed to determine whether the appropriate screening age in Africa should be even younger. In Nigeria, the average age of CRC diagnosis is between 43 and 46 years, suggesting that a screening age of 40 years may be more appropriate [22]. A lower screening age of 35 to 40 years could also be considered for those with a family history of CRC.

The Delphi Initiative Recommendations on EOCRC (DIRECt) offers the first evidence-based management guidelines for EOCRC. Although DIRECt does not mention Africa, the study includes data on African American populations. DIRECt recommends colonoscopy as the diagnostic tool for high-risk signs and symptoms of EOCRC. However, the availability of colonoscopies in Africa is limited, and where they are available, they are costly for most young people. This poses a barrier to following guidelines that recommend repeating colonoscopies every five to 10 years based on previous results [56]. Governments should reassess insurance coverage in regions where it already exists, and introduce insurance coverage in places where it is absent, to include financially burdensome procedures like colonoscopies, and explore public-private partnerships to reduce healthcare costs.

Tests like fecal immunochemical testing (FIT) and gFOBT are commonly used to evaluate CRC in Africa [61]. The DIRECt study found no significant differences in evaluation tools between EOCRC and late-onset CRC, so these tests can also be applied to EOCRC [56]. FIT and gFOBT are easy to use, and decisions should be made about which test is most suitable for specific regions. FIT is the preferred method for population-based screening, and it has advantages such as no dietary restrictions and fewer sample requirements. However, its accuracy may be a concern in hot climates, especially if proper cold storage platforms are not available [61,63]. African healthcare management teams should ensure that test kits are consistently available and stored properly to maintain the precision of FIT. Since most CRC cases in Africa are diagnosed at advanced stages, FIT should be optimized, and positive cases should be urgently referred for colonoscopy and biopsy [36]. Governments should invest in data management tools to ensure patients are not lost to follow-up.

Given that CRC can occur sporadically or due to familial or genetic reasons, it is vital to have access to germline testing tools. Where feasible, tumors should be tested for mismatch repair deficiency through microsatellite instability analysis or immunohistochemistry [56,64].

Therapeutic care

Treatment Guidelines

The DIRECt guidelines do not suggest alternative treatments for EOCRC, except in cases with pathogenic germline variants [56]. It is necessary to determine what applies to Africa since the tumor biology or metastatic pattern of CRC may differ based on location [32]. In addition, intensive treatment should be considered if EOCRC continues to be diagnosed at advanced stages in Africa. African nations can avoid the additional costs of pathogenic germline variants by ensuring environmental safety and food security, as these factors may alter the gut microbiome and cause mutations.

While there is no evidence to support different treatment approaches between early- and late-onset CRC, policymakers in each country should ensure the implementation or adaptation of the NCCN harmonized guidelines for CRC in SSA. The guideline is based on contributions from African oncology experts who determined what is feasible or peculiar to Africa based on the parent CRC guideline [34]. A lack of updated treatment guidelines for cancer has been reported as one of the challenges impeding immune checkpoint blockade usage in SSA [65]. Chemotherapeutic and immunotherapeutic medications should always be available and subsidized. Physicians should stay informed on current treatment options and be prepared to discuss them with patients, incorporating their preferences into decision-making. This consideration is significant for young adults who may be concerned about infertility risks associated with treatment [56,66]. Current evidence does not support aggressive adjuvant therapy [67]. In countries lacking a national treatment guideline, it may be a common practice for oncologists to employ treatment plans not based on protocol, with a consequential risk of aggressive adjuvant therapies [34]. It is important to note that avoiding unnecessary medications can help reduce costs and improve patient compliance. Nevertheless, physicians should be aware that chemotherapy for EOCRC may cause higher rates of nausea and vomiting, which can be managed with affordable antiemetics as adjuvants [56]. Regarding surgery, EOCRC procedures are similar to those for late-onset CRC, except in cases with additional risks [68].

Cancer Care Centers

Another challenge in EOCRC treatment is the rarity of adequate specialized cancer care centers. When established and dedicated as either EOCRC centers or broadly as adolescent and young adult cancer centers, these centers can help prevent patients from being lost to follow-up, provide specialist management for patients, and offer valuable data for cancer registries. Transportation to these centers should be subsidized to encourage attendance, particularly for young patients who may live far from these facilities [36,69,70].

Clinical Trial and Research

African nations can benefit from global clinical trials, which provide treatment opportunities for advanced disease patients and generate valuable data. Poor awareness of the symptoms of CRC, low healthcare literacy, personal beliefs, and inadequate access to treatment contribute to the late presentation of the disease and the rising mortality from EOCRC in many African countries [22]. Governments should invest heavily in local research to develop alternative and affordable diagnosis and treatment options for CRC. NGOs or consortia focused on CRC can also be integrated into research efforts and supported with adequate funding. The work of the African Research Group for Oncology (ARGO) in Nigeria can serve as a model for other African nations [71].

Post-treatment care

Post-treatment care is crucial in managing EOCRC in Africa. Many individuals aged 30 to 50 years are their families' primary wage earners. The financial burden of treatment and follow-up procedures can lead to social isolation and discourage patients from returning for care [72]. Failure to follow up can result in lost data for prospective studies and contribute to relapses, which often lead to mortality. Intensive follow-up is recommended, especially in the first five years after surgical intervention, due to the high recurrence rate after resection [56]. Governments should ensure adequate psychosocial support for EOCRC patients during and after treatment. Support groups should be tailored away from the conventional cancer support groups style to contemporary social networking events, including online meetings with other patients and EOCRC survivors [73].

Given the economic challenges faced by most African nations, increasing awareness, improving screening, and enhancing diagnosis for CRC may be a more cost-effective strategy to address the rising incidence of EOCRC and its associated mortality. Early screening and prevention can help reduce advanced disease, improve the quality of life for young adults, and preserve the productive workforce of African nations. The lack of genomic research in Africa and poor inclusion of African genome studies in genomic databases are well-known limitations for CRC screening. African genomic research has the potential to identify specific genetic drivers of EOCRC, which can pave the way for early diagnosis of CRC in young African adults. Stakeholders are encouraged to invest significantly in establishing genomic research centers in Africa, with divisions focused on studying and identifying genetic variants that correlate with an increased risk of CRC in young adults. Also, a lack of research data and literature from Africa is a significant limitation not just for this review but for healthcare experts, stakeholders, and other decision-makers who play vital roles in advancing cancer care in Africa.

## Conclusions

EOCRC is rapidly increasing across Africa, posing a major public health threat. The increase is driven by urbanization, increased popularity of western diets among Africans, sedentary lifestyles, and limited public awareness, compounded by weak health infrastructure that delays diagnosis and limits access to effective screening and treatment for younger populations.

To effectively combat EOCRC in Africa, a multidimensional and coordinated strategy must be urgently implemented. First, widespread awareness campaigns targeting young populations are essential to promote understanding of risk factors, symptoms, and the importance of early screening. To maximize reach, these efforts should involve mass media, schools, digital platforms, and community engagement. Second, existing screening guidelines must be revised to reflect the shifting epidemiology and lower the recommended starting age to at least 45 years or earlier for high-risk individuals. Affordable and feasible tools such as FIT should be scaled up for population-level screening, supported by investments in diagnostic infrastructure like colonoscopy services and pathology labs. Treatment should be guided by resource-sensitive protocols such as the NCCN harmonized guidelines tailored to the realities of African healthcare systems. Insurance schemes and public-private partnerships must also address financial barriers to care. Importantly, post-treatment care should include psychosocial, fertility, and survivorship support, especially given the age and life stage of EOCRC patients. Establishing specialized EOCRC or young adult cancer centers can facilitate comprehensive care and improve outcomes. Investment in local research and clinical trials is also vital to generate data-driven, culturally appropriate interventions. Without decisive action, Africa risks losing a growing number of its young, productive population to a preventable and treatable disease.
